# Evaluation of the effects of the T-type calcium channel enhancer SAK3 in a rat model of TAF1 deficiency

**DOI:** 10.1016/j.nbd.2020.105224

**Published:** 2020-12-24

**Authors:** Chinnasamy Dhanalakshmi, Udaiyappan Janakiraman, Aubin Moutal, Kohji Fukunaga, Rajesh Khanna, Mark A. Nelson

**Affiliations:** a Department of Pathology, University of Arizona College of Medicine and College of Pharmacy, Tucson, AZ, USA; b Department of Pharmacology, University of Arizona College of Medicine and College of Pharmacy, Tucson, AZ, USA; c The Center for Innovation in Brain Sciences, The University of Arizona Health Sciences, Tucson, AZ, United States; d The BIO5 Institute, University of Arizona, United States; e Department of Pharmacology, Graduate School of Pharmaceutical Sciences, Tohoku University, Sendai, Japan

**Keywords:** TAF1, Intellectual disability syndrome, SAK3, CaV3.1, Cerebral cortex

## Abstract

The TATA-box binding protein associated factor 1 (TAF1) is part of the TFIID complex that plays a key role during the initiation of transcription. Variants of TAF1 are associated with neurodevelopmental disorders. Previously, we found that CRISPR/Cas9 based editing of the TAF1 gene disrupts the morphology of the cerebral cortex and blunts the expression as well as the function of the CaV3.1 (T-type) voltage gated calcium channel. Here, we tested the efficacy of SAK3 (ethyl 8′-methyl-2′, 4-dioxo-2-(piperidin-1-yl)-2′H-spiro [cyclopentane-1, 3′-imidazo [1, 2-a] pyridine]-2-ene-3-carboxylate), a T-type calcium channel enhancer, in an animal model of TAF1 intellectual disability (ID) syndrome. At post-natal day 3, rat pups were subjected to intracerebroventricular (ICV) injection of either gRNA-control or gRNA-TAF1 CRISPR/Cas9 viruses. At post-natal day 21, the rat pups were given SAK3 (0.25 mg/kg, p.o.) or vehicle for 14 days (i.e. till post-natal day 35) and then subjected to behavioral, morphological, and molecular studies. Oral administration of SAK3 (0.25 mg/kg, p.o.) significantly rescued locomotion abnormalities associated with TAF1 gene editing. SAK3 treatment prevented the loss of cortical neurons and GFAP-positive astrocytes observed after TAF1 gene editing. In addition, SAK3 protected cells from apoptosis. SAK3 also restored the Brain-derived neurotrophic factor/protein kinase B/Glycogen Synthase Kinase 3 Beta (BDNF/AKT/GSK3β) signaling axis in TAF1 edited animals. Finally, SAK3 normalized the levels of three GSK3β substrates - CaV3.1, FOXP2, and CRMP2. We conclude that the T-type calcium channel enhancer SAK3 is beneficial against the deleterious effects of TAF1 gene-editing, in part, by stimulating the BDNF/AKT/GSK3β signaling pathway.

## 1. Introduction

T-type calcium (CaV3) channels are low voltage-activated calcium channels that transiently open to evoke tiny Ca^2+^ currents (reviewed in (Perez-Reyes, 2003)). T-type calcium channels regulate calcium influx (Cazade et al., 2017), and also activate calcium-induced calcium release from internal calcium source (Kitchens et al., 2003; Coulon et al., 2009). These observations indicate a key function for T-type calcium channels in regulating intracellular calcium homeostasis and maintaining cellular functions (Assandri et al., 1999; Chemin et al., 2000; Cazade et al., 2017).

T-type calcium channels consist of three subtypes (CaV3.1, CaV3.2, and CaV3.3) with unique functions (Iftinca and Zamponi, 2009). In proliferative cells such as cancer cells, adipocytes, and stem cells, T-type calcium channels participate in cellular proliferation and cell cycle (Sallan et al., 2018; Rodman et al., 2005; Oguri et al., 2010). Recently, interest in the role of T-type calcium channels in the brain has increased because of their importance in neurodevelopmental disorders such as autism spectrum disorder, neural tube defects, and TATA-box binding protein associated factor 1 (TAF1) intellectual disability (ID) syndrome (Cheong and Shin, 2013; Abdul-Wajid et al., 2015; Janakiraman et al., 2019).

(Yabuki et al., 2017b) recently developed, a novel therapeutic candidate, SAK3 (ethyl 8′-methyl-2′,4-dioxo-2-(piperidin-1-yl)-2′H-spiro[cyclopentane-1,3′-imidazo[1,2-a] pyridine]-2-ene 3 carboxylate). SAK3 stimulates Ca^2+^ entry into neurons via CaV3.1 and CaV3.3 T-type channel. SAK3 treatment prevents cognitive impairment in methimazole-induced hypothyroidism and in brain ischemia induced by nicotinic acetylcholine receptor stimulation (Husain et al., 2018; Yabuki et al., 2017a). SAK3 inhibited amyloid beta (Aβ) accumulation and aggregation in amyloid precursor protein (APP) transgenic mice and elicits an anti-depressant action on olfactory bulbectomized (OBX) mice (Xu et al., 2018). Together, SAK3-mediated rescue of cognitive impairment, likely via its activation of CaV3.1, make this small molecule an attractive drug candidate for ameliorating symptoms of TAF1 ID syndrome.

We have previously shown that TAF1 gene editing alters the morphology of the cerebral cortex (Janakiraman et al., 2019). Our group and others have also shown that TAF1 gene editing decreases the expression and function of the CaV3.1 T-type calcium channel in vitro and in vivo (Hurst et al., 2018; Gudmundsson et al., 2019; Janakiraman et al., 2019). We also showed that SAK3 treatment reverses the loss of purkinje cells after TAF1 gene editing. (Janakiraman et al., 2020). Therefore, here we investigated the potential beneficial effects of SAK3 treatment on the cerebral cortex. Within each cortical layer, there are several subtypes of pyramidal neurons. We chose to analyze somatosensory motor cortex pyramidal layer 5 for analysis because of their large soma size and broad-tufted cells of the motor cortex (Jiang et al., 2020). We also provide new insights into the signaling of SAK3 in the central nervous system.

## 2. Materials and methods

### 2.1. Animals

Pathogen-free, normal E18 pregnant Sprague–Dawley rats (Envigo Laboratories) were housed 1 per cage in temperature- (23 ± 3 °C) and light (12-h light/12-h dark cycle; lights on 07:00–19:00)-controlled rooms with standard rodent chow and water available ad libitum. The neonates are designated as post-natal day 0 (PD0) on the day of birth, the litter size range included in the current study was 11–14 pups.

**Fig. 1.**
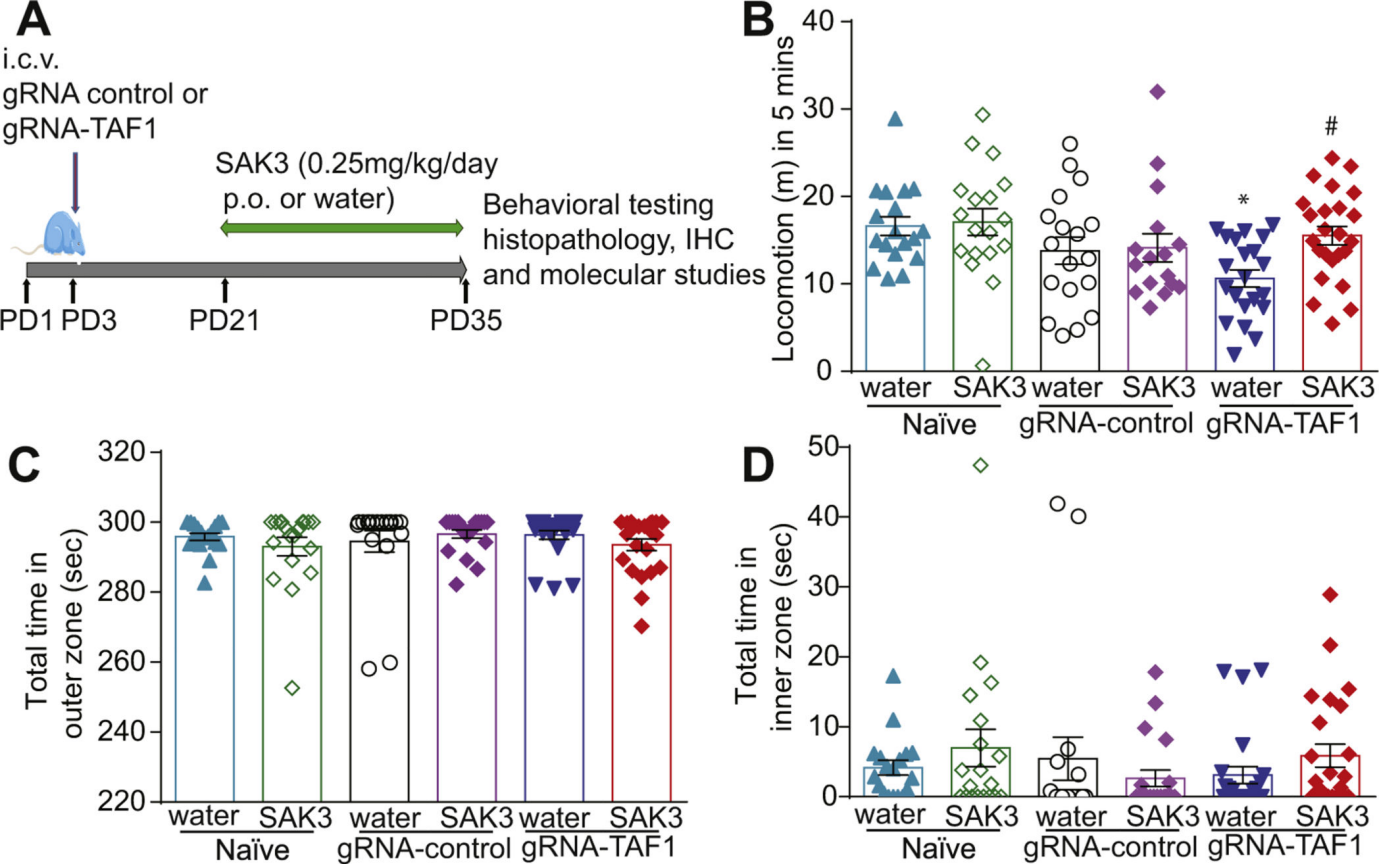
Experimental design for these studies and behavioral assessment of motor function and anxiety. (A) Experimental design for behavioral, morphologic, and molecular studies. (B) The TAF1-edited animals displayed decreased locomotion relative to naïve and gRNA-control group animals. (C & D) No differences in thigmotaxis was observed between the experimental groups. Data are shown as mean ± S.E.M., *n* = 21 to 24 per experimental condition. *p < 0.05 versus; naïve, #p < 0.05 versus gRNA-control (ANOVA followed by Tukey’s test). The experiments were conducted in an investigator blinded manner.

After weaning the rat pups were separated from the dams and maintained 4 per cage. Animals were divided into six groups. SAK3 (Catalog No: SML-2039–5MG, Sigma Aldrich) was dissolved in distilled water and orally administered (0.25 mg/kg, p.o) to the animals from postnatal day 21 to 35 (PD21–35) (Fig. 1A). We chose to administer this dose of SAK3 because it has been shown to be effective in restoring cognitive function in a mouse model of Alzheimer’s disease (Wang et al., 2018). Rats were sacrificed at PD35. Animals were behaviorally assessed before being euthanized for histological, immunostaining and protein expression analysis. All biochemical and behavior experiments were performed in a blinded fashion. Animal protocols were approved by the Institutional Animal Care and Use Committee of the College of Medicine at the University of Arizona and conducted in accordance with the Guide for Care and Use of Laboratory Animals published by the National Institutes of Health.

### 2.2. CRISPR/Cas9-mediated targeting of TAF1 gene

Our strategy to truncate TAF1 focused on targeting exon 1 of the TAF1 gene using a guide RNA (gRNA) has been described previously (Moutal et al., 2017; Moutal et al., 2018a,b; Sandweiss et al., 2018). We targeted this exon to ensure total removal of the TAF1 protein. Using this approach, we expect minimal to none off-target activity of the Cas9 enzyme as we and others verified before (Moutal et al., 2017; Ma et al., 2017). The gRNA sequence (GTGTCTGACATGACGGCGGA, quality score 94) was inserted into the restriction site of the pL-CRISPR.EFS. tRFP lenti-plasmid (Cat#57819, Addgene, Cambridge, MA) (Heckl et al., 2014), a plasmid that allows for simultaneous expression of (i) the Cas9 enzyme; (ii) the gRNA; and (iii) a red fluorescent protein (tRFP) – to control for transduction efficiency. All plasmids were verified by Sanger sequencing (Eurofins, Louisville, KY). Lenti-viral plasmids were packaged in lentiviruses by Viracore (USCF, CA) at titers routinely above 10^7^ infectious particles per ml.

### 2.3. Intracerebroventricular injections

Bilateral intracerebroventricular (ICV) injections were performed as previously described in Sprague-Dawley (SD) rat pups on postnatal day 3 (Delenclos et al., 2017; Pang et al., 2003). Briefly, newborn SD rat puts were anesthetized by isoflurane. A 10 μl syringe (Hamilton Gastight Syringe, #1701) was used to pierce the skull (coordinates from bregma: − 0.6 mm posterior, ± 1.75 mm lateral/medial, and − 2.5 mm ventral), and 2.5 μl of CRISPR/Cas9 lentivirus (gRNA-control or gRNA-TAF1) was injected into each cerebral ventricle. Previous studies demonstrated that this route of delivery allows for efficient reduction of TAF1 expression in all cells of the cerebellum, cerebral cortex and hippocampus (Janakiraman et al., 2019). Neonatal rat pups were kept with the dam until weaned.

### 2.4. Open field test

Open field test is used for analyzing the behavior and locomotion in rats. The apparatus is 120-cm diameter circular arena, bordered by a 50-cm-high wall made of wood. The floor of this chamber was divided into central and peripheral zone. The rat pups were placed into the peripheral zone of an open field chamber and locomotion was observed for 5 min (Rajasankar et al., 2009).

### 2.5. Nissl staining

After dehydration of the tissue with 30% sucrose, 20 μm sections were cut, stained with cresyl violet dye (Catalog No: C5042–10G, Sigma), and mounted with Richard-Allan scientific mounting medium (Catalog No: 4112, Thermo Scientific) then visualized the somatosensory motor cortex pyramidal layer 5 of motor cortex.

### 2.6. Cell counting

Twelve visual fields (0.6 mm2) of the somatosensory motor cortex pyramidal layer 5 of the motor cortex were randomly imaged from each section. The number of stained cells in each field was counted at a 40× magnification. Data were expressed as the number of cells per field (Liu et al., 2011).

### 2.7. TUNEL assay

Cerebral cortex tissue sections of 20 μm were cut and 5–7 sections chosen according to systematic random sampling scheme from each sample were processed with In Situ Cell Death Detection Kit, Fluorescein (Catalog No: 11684795910; Roche, Millipore Sigma, USA) according to the manufacturers protocol for tissues. Then we focused on the somatosensory motor cortex pyramidal layer 5 of the motor cortex using a 20× objective and apotome 2(LSM510, Carl Zeiss).

### 2.8. Immunohistochemistry

The antibody used in our studies are listed in Table 1. Rats (*n* = 6 animals per experimental condition) were perfused with saline and 4% formaldehyde in Phosphate buffer saline (PBS) at PD35, and the brains were extracted and post-fixed in 4% paraformaldehyde for 8 h at 4 °C. Cerebral cortex sections were cut sagittal at 20 μm using a cryostat (Microm HM 505 E). After rinsing the sections in PBS for 5 min, the sections were incubated with a 0.1% H2O2 solution in PBS for 5 min, rinsed in PBS for 5 times for 5 min, and incubated for 30 min with 0.4% Triton X-100, rinsed in PBS and blocked with 8% goat serum, and 1% Triton X-100 in PBS. After blocking, the sections were incubated at 4 °C for overnight with the indicated antibodies diluted in 4% goat serum in PBS. The sections were washed in 1% goat serum in PBS, incubated with secondary antibody anti-rabbit Alexa fluor 488 (Life Technologies) or anti-mouse Alexa fluor 488 (Life Technologies), as needed, in 4% goat serum for 2 h, washed with PBS 3 times for 5 mins, and incubated with DAPI (Catalog No: D1306, Thermofisher Scientific) at concentration of 50 ng/ml for 2 mins. Sections were then washed and further air dried, and cover slipped with glycerol. All procedures were performed at room temperature. Stained slides were observed under a fluorescence microscope, focusing on the somatosensory motor cortex pyramidal layer 5 using a 20× objective and apotome 2 (LSM510, Carl Zeiss). Immunflourescence was quantified in 12 different fields from 4 different animals per experimental as previously described (Janakiraman et al., 2019).

### 2.9. Western blotting analysis

After decapitation of animals, cerebral cortex was dissected from the whole brain tissue, snap frozen in liquid nitrogen prior to storage at − 80 °C until analysis. Western blot analyses were performed as described (Janakiraman et al., 2019). In brief, frozen samples were homogenized with RIPA buffer and centrifuged at 4 °C, 20,000 ×g for 20 min. Supernatant protein concentrations were determined using BCA method (Catalog No: #23227, Thermo Scientific), and samples were then boiled 5 min in Laemmli’s sample buffer (Catalog No: NP0008, Life Technologies). Equal amount of protein was loaded and run on SDS-polyacrylamide gels (Catalog No: #4568084, Bio-Rad) and then transferred to polyvinylidene difluoride membrane (Catalog No: #1620177, Bio-Rad). After transfer, membranes were blocked with TBST solution (50 mM Tris-HCl, pH 7.5, 150 mM NaCl, and 0.1% Tween 20) containing 5% non-fat dry milk at 4 °C for 1 h. After blocking membranes were incubated overnight at 4 °C with anti-cleaved caspase-3 (Asp175) (1:1000; Cell Signaling), anti-Bax (1:1000; Cell Signaling), anti-Bcl-2 (1:1000; Abcam), anti-phospho-GSK3β (Ser9) (1:1000; Cell Signaling), anti-GSK3β (1:1000; Cell Signaling), anti-BDNF (1:750; Abcam), anti-Foxp2 (1:1000; Abcam), anti-CRMP2 pThr509/Thr514 (1:1000; Kina Source Limited), anti-CRMP2 (1:1000; Sigma) and βIII-Tubulin (1:1000; Promega) in 3% BSA. After washing, membranes were incubated with secondary antibody diluted in 3% BSA. Blots were developed using an ECL detection system (Catalog No: 20–300B, Prometheus Protein Biology Products, USA) and signals were quantified using Image Studio Digits software version 5.2 (Li-Cor).

### 2.10. Statistics

All data was first tested for a Gaussian distribution using a D’Agostino-Pearson test (Graphpad Prism 8 Software, Graphpad, San Diego, CA). The statistical significance of differences between means was determined using a parametric ANOVA followed by Tukey’s post-hoc test or a non-parametric Kruskal Wallis test followed by Dunn’s post-hoc test depending on if datasets achieved normality. Differences were considered significant if *p* ≤ 0.05. Error bars in the graphs represent mean ± SEM.

## 3. Results

### 3.1. SAK3 treatment improves locomotion

We used the Open Field Test to measure locomotor activity and anxiety-like behavior in the animals. We found that TAF1 edited animals displayed decreased locomotion compared to the controls and SAK3 treatment increased locomotion of TAF1 edited animals (Fig. 1B). We also measured thigmotaxis, a parameter of anxiogenic behavior in rodents (Seibenhener and Wooten, 2015). We found no difference in thigmotaxis between the experimental groups (Fig. 1C&D).

### 3.2. SAK3 treatment attenuates the morphological abnormalities caused by TAF1 deletion

**Fig. 2.**
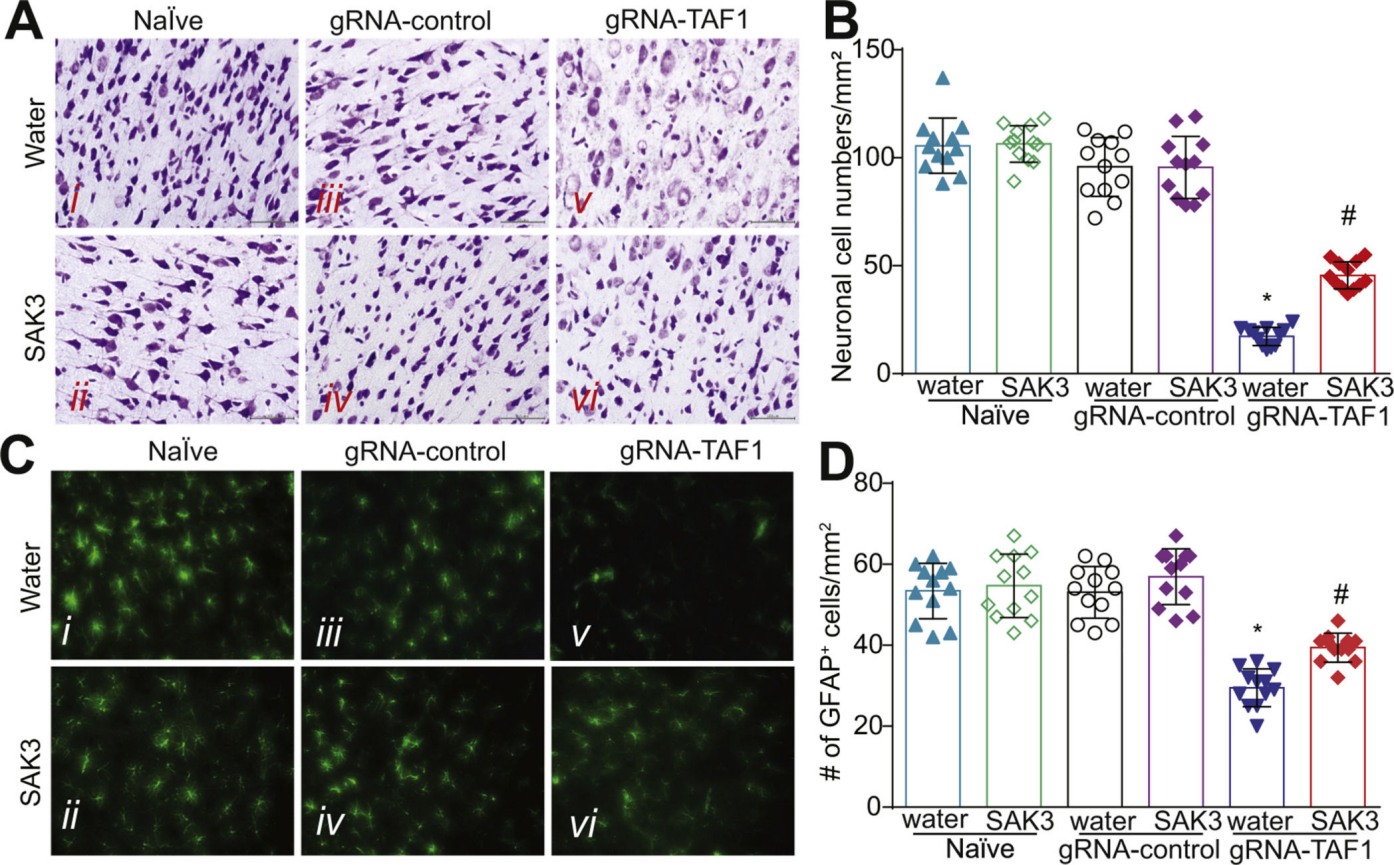
SAK3 treatment improves neuronal morphology and causes glial cells activation in the cerebral cortex of TAF1 gene edited rats. (A) The morphology of the cerebral cortex was evaluated by Nissl Staining. The Nissl staining showed shrunken neurons with vacuolated intercellular spaces and many unstained regions of the somatosensory motor cortex pyramidal neurons in layer 5 after TAF1 gene editing. SAK3 administration to the TAF1 edited animals improved the morphology of the cortical neurons. (B) Enumeration of the cells in each treatment group. Note decreased cell viability in TAF-1 edited animals compared to the control groups and animals treated with SAK3. (C) Expression of GFAP was decreased in TAF1-edited animals as compared to naïve and CRISPR-control groups. SAK3 administration to the TAF1 edited animals shows increased the number of GFAP positive cells. (D) Number of GFAP positive cells in each of the experimental conditions. Data are shown as mean ± S.E.M., *n* = 12 fields per animal, 4 animals per experimental condition. *p < 0.05 versus; naïve, #p < 0.05 versus gRNA-control (ANOVA followed by Tukey’s test). Scale bars: 200 μm. The experiments were conducted in a blinded fashion.

We previously documented that TAF1-editing caused morphological abnormalities in the cells of the cerebral cortex (Janakiraman et al., 2019), so we evaluated the morphology of the neurons in layer 5 of the motor cortex by Nissl staining. Our histopathology analysis showed that neurons in the control group were arranged tightly with regular pyramidal morphology at layer 5 within the sensory-motor cortex and had an intact cell structure. The blue Nissl bodies in the neurons were visible and distinct. As we previously reported, the morphology of the neurons in the motor cortex at layer 5 was clearly different in TAF1-edited animals. The shape of the neurons changed from a pyramidal to a stellate morphology and many neurons appeared to be vacuolated with enlarged intercellular spaces. Also, the neuronal cells did not stain as distinctly as control neuronal cells with the Nissl dye (Fig. 2A). However, SAK3 administration improved the morphology of the neurons in cortex (Fig. 2A). In TAF1-edited animals, we found a drastically reduced number of neurons compared to Naïve or gRNA-control rats (Fig. 2B). SAK3 administration resulted in an increased number of neurons compared to TAF1-edited animals (Fig. 2B). Treatment with SAK3 had no effect on the Naïve and gRNA-control group compared to the vehicle (water) (Fig. 2B). Collectively, these results strongly suggest that TAF1 is directly implicated in neuronal survival.

Among the essential cell types in the brain, astrocytes are the most abundant population. Astrocytes retain proliferative capacity, and their functions are crucial for neuronal survival (Jakel and Dimou, 2017). Astrocytes are critical for mediating ion homeostasis, growth factor responses and neurotransmitter functions in the brain (Devinsky et al., 2013). We previously reported a decrease in GFAP-positive astrocytes within the granular layer of the cerebellum in TAF1-edited animals (Janakiraman et al., 2019). Similarly, we found decreased numbers of astrocytes in the cerebral cortex of TAF1-edited rats treated with SAK3 (Fig. 2C, D). Thus, morphological alterations of neurons and astrocytes induced by TAF1 editing in layer 5 of the cerebral cortex can be reversed by SAK3 treatment.

### 3.3. Administration of SAK3 attenuates apoptosis

**Fig. 3.**
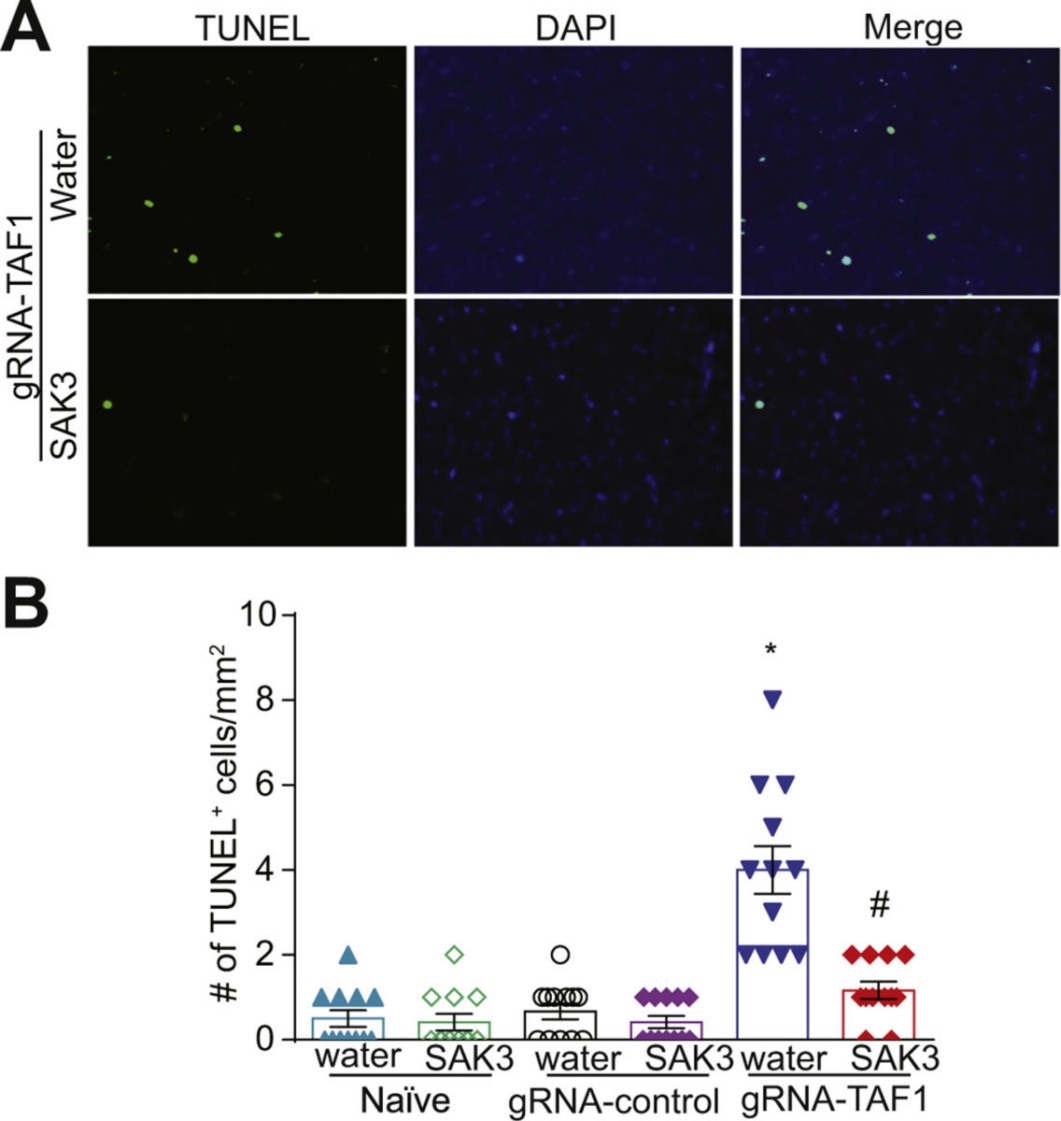
SAK3 treatment protects against apoptosis caused by TAF1 gene editing in the cerebral cortex. Apoptosis was assessed in somatosensory motor cortex pyramidal neurons in layer 5 samples using TUNEL assay. (A) Shown are photomicrographs from gRNA-TAF1 edited animals and gRNA-TAF1 edited animals treated with SAK3. The data from the control groups can be found in the Supplementary data. Note SAK3 reduced the number of TUNEL positive neuronal cells in gRNA-TAF1 edited animals. (B) Summary of the number of TUNEL positive cells in each of the experimental conditions. Data are shown as mean ± S.E.M., n = 12 fields per animal, 4 animals per experimental condition. *p < 0.05 versus; naïve and gRNA-TAF1 = SAK3 group (ANOVA followed by Tukey’s test). Scale bars: 200 μm. The experiments were conducted in an investigator-blinded manner.

**Fig. 4.**
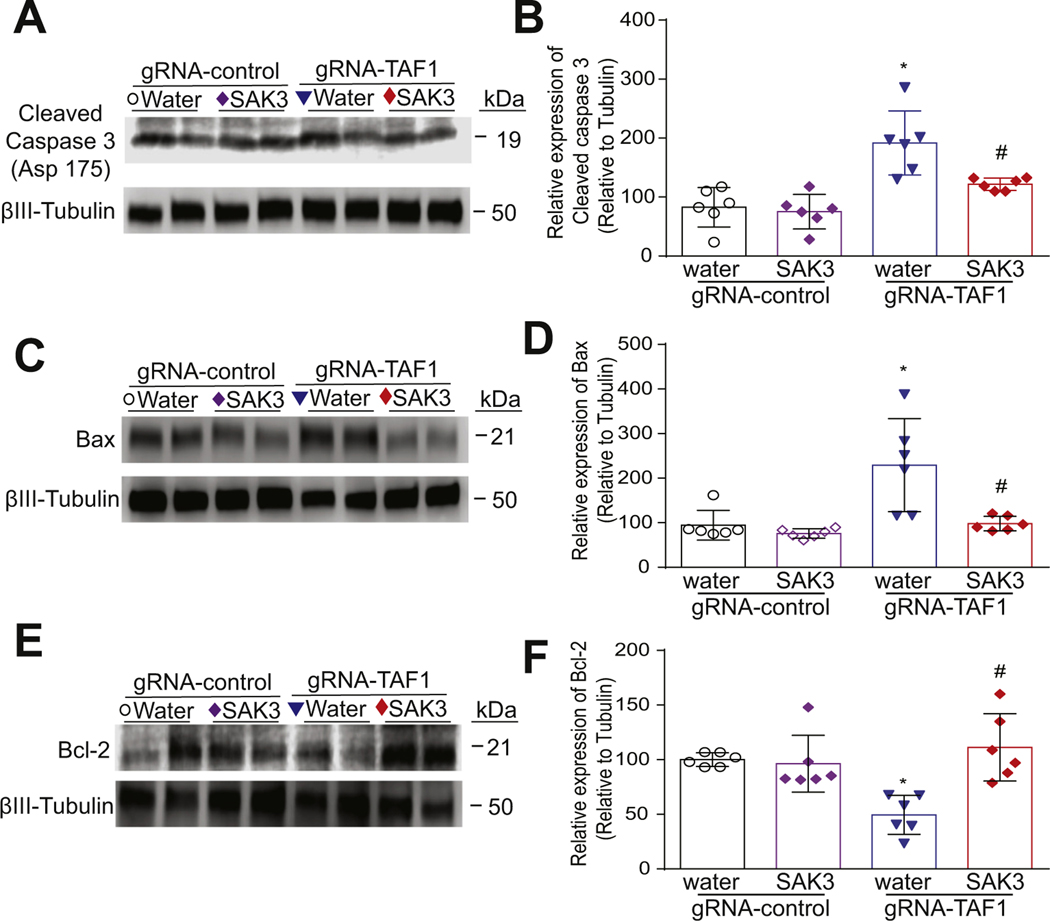
The effects of SAK3 treatment on regulators of apoptosis in the cerebral cortex. (A) The effects of SAK3 administration on cleaved caspase 3 levels. Representative Western analysis is shown from cerebral cortex samples from animals from each of the experimental conditions. Note the increased cleaved caspase 3 in gRNA-TAF1 edited animals and diminished cleaved caspase 3 levels in gRNA-TAF1 SAK3 treated animals. (C) Blots show the increased BAX levels in gRNA-TAF1 edited animals, whereas SAK3 reduced BAX levels to control levels. (E) Blots show decreased Bcl-2 levels in gRNA-TAF1 edited animals, whereas SAK3 increase Bcl-2 expression to control levels. (B, D, F) Quantification of Western analysis from independent experiments. Data shown are mean ± SEM, *n* = 6 animals per each experimental condition. *p < 0.05 versus gRNA-control water and RNA-control SAK3 group; # p < 0.05 versus gRNA-TAF1 water group (ANOVA followed by Tukey’s test).

Because we observed morphological abnormalities and decreased cell viability in TAF1 edited animals which was rescued by SAK3, we assessed if apoptosis could be enhanced in TAF1 edited animals. We observed an increase in terminal deoxynucleotidyl transferase dUTP nick end labeling (TUNEL) positive cells in layer 5 of the cerebral cortex in TAF1 edited animals. SAK3 treatment reduced the number of TUNEL positive cells to control levels in TAF1 edited animals (Fig. 3A, B). In agreement with our previous observations (Janakiraman et al., 2020), no significant changes in the TUNEL positive cells of water and SAK3 treatment to the naïve and gRNA-control animals were observed. The protective effects of SAK3 against apoptosis was corroborated by (i) the down-regulation of activated caspase 3(Fig. 4A, B); (ii) the down-regulation of the pro-apoptotic factor BCL2 Associated X (BAX), an apoptosis regulator (Fig. 4C, D); and (iii) up-regulation of B-cell lymphoma 2 (Bcl-2), an apoptosis suppressor gene (Fig. 4E, F).

### 3.4. SAK3 treatment stimulates Brain-derived neurotrophic factor/protein kinase B/Glycogen Synthase Kinase 3 Beta (BDNF/AKT/GSK3β) signaling in the cerebral cortex of TAF-1 edited animals

**Fig. 5.**
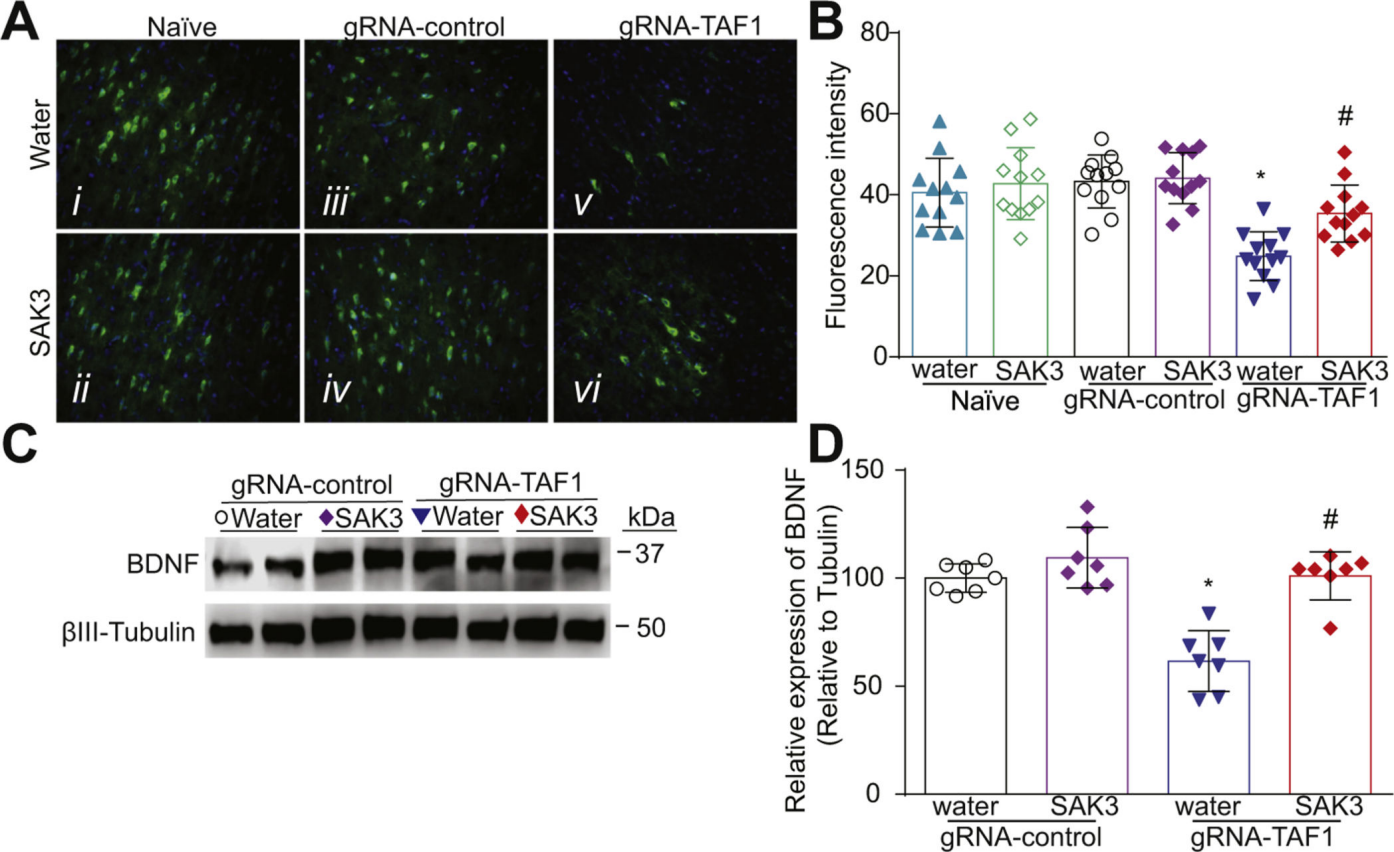
The effects of SAK3 treatment on BDNF expression in the cerebral cortex of TAF1 edited animals. (A) Expression of BDNF was decreased in TAF1-edited animals (Av) as compared to all other experimental groups (Ai-iv). SAK3 administration to the TAF1 edited animals shows increased number of BDNF-positive cells as compared to TAF1 edited group (Avi & v). (C) Representative Western analysis of BDNF is shown from cerebral cortex samples from animals from each of the experimental conditions. Note decreased BDNF levels in gRNA-TAF1 edited animals, whereas SAK3 increases BDNF expression to control levels. (B) Summary of BDNF expression, Data are shown as mean ± S.E.M., n = 12 fields per animal, 4 animals per experimental condition. Scale bars: 200 μm. (D) Quantification of Western analysis from three independent experiments. Data shown are mean ± SEM, n = 6 animals per each experimental condition. *p < 0.05 versus; naïve and gRNA-TAF1 = SAK3 group (ANOVA followed by Tukey’s test). The experiments were conducted in an investigator-blinded manner.

**Fig. 6.**
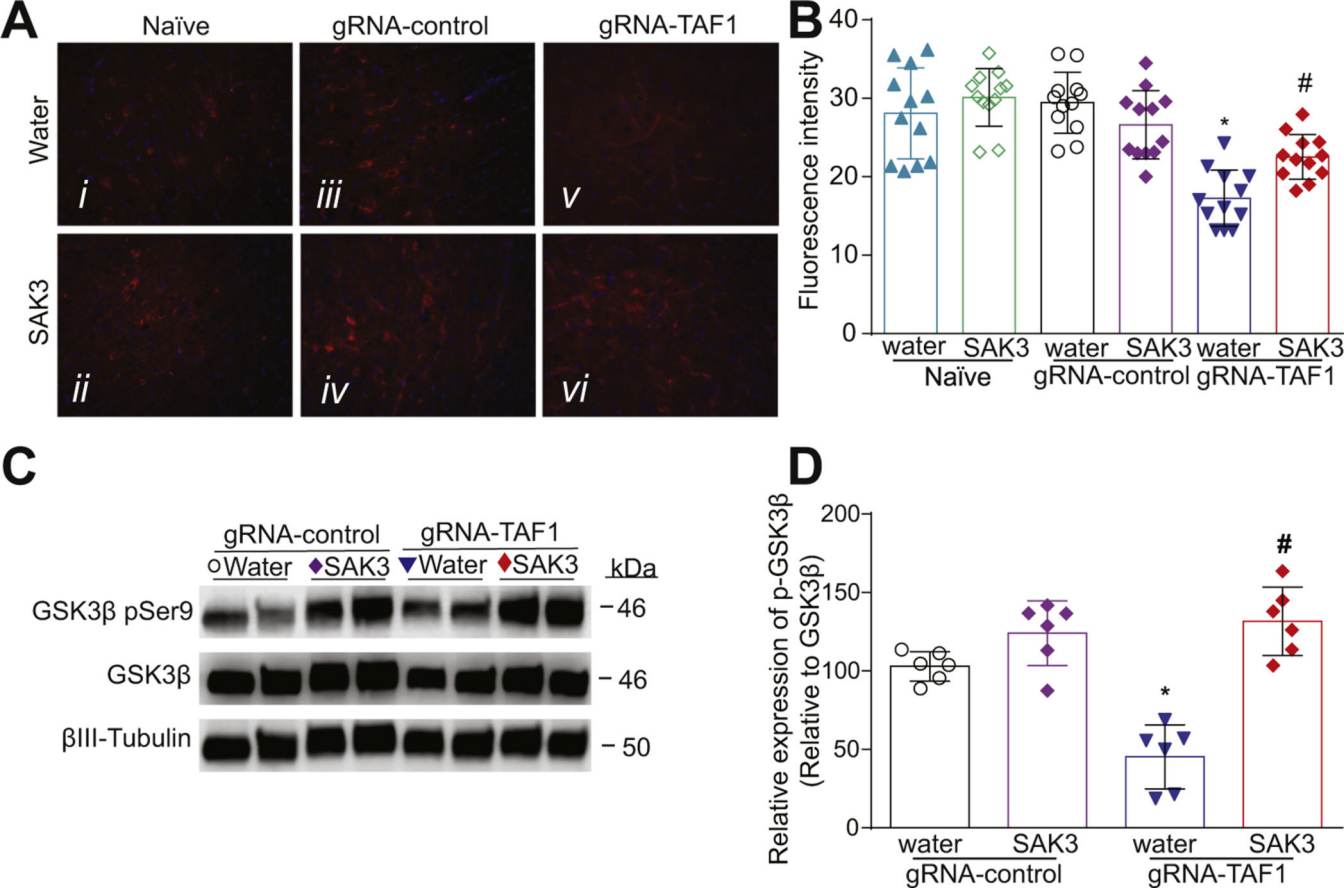
The effects of SAK3 treatment on AKT/GSKβ signaling in the cerebral cortex of TAF1 edited animals. (A) Expression of p-AKT was decreased in TAF1-edited animals (Av) as compared to all other experimental groups (Ai-iv). SAK3 administration to the TAF1 edited animals shows increased expression of p-AKT as compared to TAF1 edited group (Avi & v). (C) Representative Western analysis of p-GSK3β is shown from cerebral cortex samples from animals from each of the experimental conditions. Note decreased p-GSK3β levels in gRNA-TAF1 edited animals, whereas SAK3 increases p-GSK3β expression to control levels. (B) Summary of p-AKT expression, Data are shown as mean ± S.E.M., n = 12 fields per animal, 4 animals per experimental condition. Scale bars: 200 μm. (D) Quantification of Western analysis from three independent experiments. Data shown are mean ± SEM, n = 6 animals per each experimental condition. *p < 0.05 versus; naïve and gRNA-TAF1 = SAK3 group (ANOVA followed by Tukey’s test). The experiments were conducted in an investigator-blinded manner.

We examined the BDNF/AKT signaling pathway because previous studies demonstrated that BDNF can protect cortical neurons from apoptosis (Poser et al., 2003). Consequently, we investigated whether SAK3 treatment could be neuroprotective through this pathway. We observed that BDNF levels were decreased in TAF1 edited animals compared to naïve animals or injected with gRNA-control CRISPR. SAK3 administration restored BDNF expression to the levels seen in naïve and gRNA-control groups (Fig. 5A-D). Protein kinase B (AKT) phosphorylation at Ser 473 is associated with its activation (Aneichyk et al., 2018). We found that activated AKT (phosphorylated at Ser 473) was decreased in the cortex of TAF-1 edited animals which was prevented by SAK3 treatment (Fig. 6A, B). Glycogen synthase kinase 3 (GSK3) has two isoforms, GSK3α and GSK3β, that are inactivated by AKT phosphorylation on Ser 21 and Ser 9, respectively (Cross et al., 1995). Inhibition of GSK3β is known to protect against apoptosis in many situations (Pap and Cooper, 1998). GSK3β is a downstream target of AKT and activation of AKT inhibits GSK3β by inducing its phosphorylation (Wu et al., 2007). We found that phospho (Ser 9)-GSK3β levels were decreased in TAF1 edited animals and SAK3 treatment increased phopho-GSK3β levels (Fig. 6C, D). Similar effects were seen in the cerebellum (Suppl. Fig. 2).

**Fig. 7.**
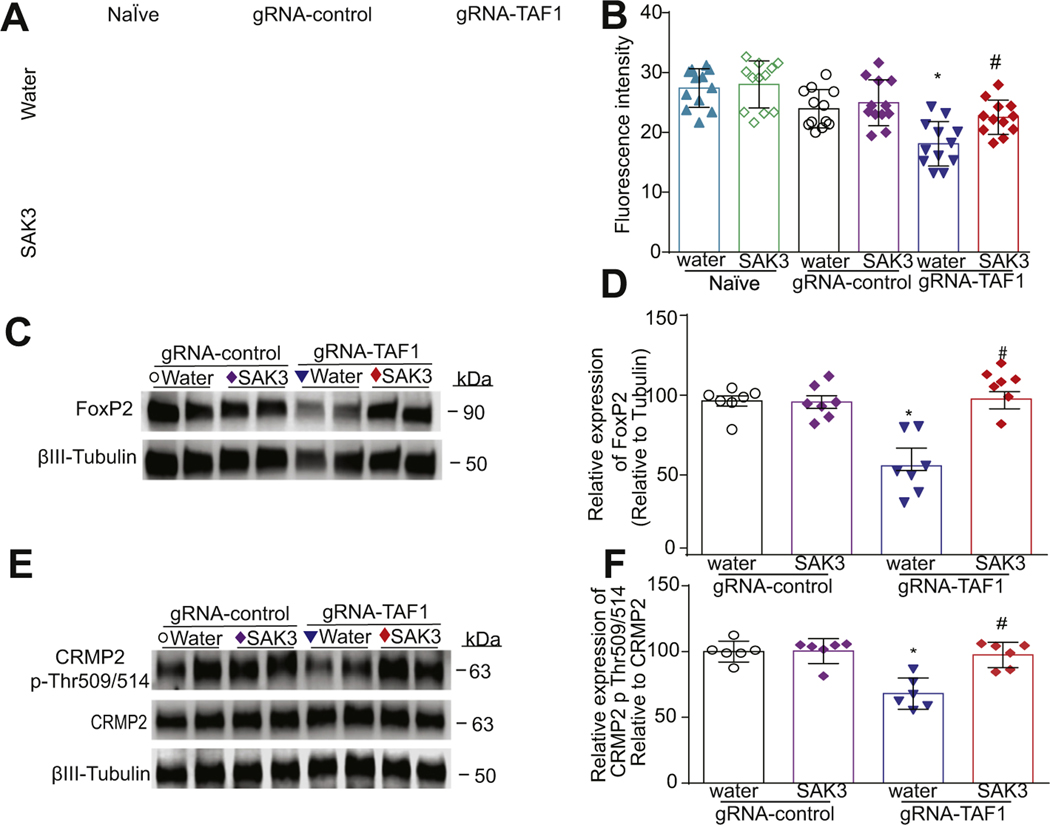
The effects of SAK3 treatment on the expression of GSK3β substrates. (A) TAF-1 editing decreased CaV3.1 T-type channel expression which was restored by SAK3 treatment in somatosensory motor cortex pyramidal neurons in layer 5. (B) Summary of CaV3.1 expression, Data are shown as mean ± S.E.M., n = 12 fields per animal, 4 animals per experimental condition. Scale bars: 200 μm. (C) FOXP2 expression was markedly reduced in gRNA TAF1 edited animals and again normalized by SAK3 treatment. (D) Summary of data shown in panel C. (E) p-CRMP2 levels were suppressed in gRNA TAF1 edited animals. However, SAK3 returned the phosphorylation levels of CRMP2, at Thr514 to control levels. (F) Quantification of Western analysis from three independent experiments. Data shown are mean ± SEM, n = 6 animals per each experimental condition. *p < 0.05 versus gRNA-control water and RNA-control SAK3 group; # p < 0.05 versus gRNA-TAF1 water group (ANOVA followed by Tukey’s test).

GSK3 is a highly evolutionarily conserved multifaceted ubiquitous enzyme with over 40 different downstream substrates (Wildburger and Laezza, 2012; Jaworski et al., 2019). We next evaluated three proteins known to be regulated by GSK3β – (i) (CaV3.1, (ii) the transcription factor Forkhead box protein P2 (FOXP2), and (iii) the axonal guidance and growth cone collapsin response mediator protein 2 (CRMP2)(Chew and Khanna, 2018; Moutal et al., 2019; Khanna et al., 2012). All three are expressed and have functions in cortical neurons (Morgan-Smith et al., 2014; Bonkowsky et al., 2008; Wildburger and Laezza, 2012; Cheong and Shin, 2013). In agreement with our previous findings (Janakiraman et al., 2019), we found a decrease in CaV3.1 expression in TAF1 edited animals which was reversed by SAK3 treatment (Fig. 7A, B). We observed a decrease of FOXP2 protein levels in TAF1-edited animals which was restored by SAK3 treatment (Fig. 7C, D). Accordingly, while CRMP2 expression was unchanged, its phosphorylation by GSK3β (at T509/T514) was decreased in TAF1 edited animals and rescued by SAK3 treatment (Fig. 7E, F).

## 4. Discussion

In the present study, we demonstrated that the T-type channel enhancer SAK3 can efficiently reverse behavioral, morphological, and biochemical defects induced by TAF1 editing in neonatal rats. Pyramidal neurons in layer 5 in the sensory-motor cortex can integrate a large amount of information and propagate signals because of the large dendritic tree and the lengths of their axons; additionally, they are the output neurons of the cerebral cortex and the main source of cognitive and motor development (Jiang et al., 2020). Based on these findings we made the following salient observations: (a) SAK3 treatment improves locomotion defects associated with TAF1 gene editing; (b) SAK3 treatment reduces the number of cortical neurons and astrocytes within the cerebral cortex that were lost following TAF1 editing induced apoptosis; and (c) SAK3 treatment re-activates BDNF/AKT/GSK3β signaling in TAF1 edited animals.

The cerebral cortex and the cerebellum are both involved in movement and there are reciprocal interconnections by the cerebello-thalamo-cortical pathways (Mendoza and Merchant, 2014). The motor cortex is the region within the cerebral cortex important for planning, control, and execution of voluntary movements. In agreement with our previous findings, we observed abnormalities in the somatosensory motor cortex pyramidal neurons in layer 5(Janakiraman et al., 2019). The somatosensory motor cortex pyramidal neurons in layer 5 are involved in motor signaling and cognitive development. The cerebellum has traditionally been viewed as a brain region involved in motor control, since it receives all the information from the motor cortex, sensory cortex, and spinal cord. However, we present findings that the abnormalities within the cerebral cortex could also contribute to the impairment locomotion seen in gRNA-TAF1 edited animals.

Consistent with our previous findings in the cerebellum, we found that TAF1 editing decreased the number of GFAP positive astrocytic cells (Janakiraman et al., 2019). Astrocytes play essential roles in regulating synaptic connectivity and function during development and in the adult. In development, astrocytes regulate synapse number by secreting synaptogenic signals, such as thrombospondins, and the matricellular proteins hevin, and glypicans, which play a role synapse formation (Allen et al., 2012; Christopherson et al., 2005; Kucukdereli et al., 2011) and by eliminating excess synapses by the phagocytosis receptors MER/AXL/TYRO3 receptor kinase family (MERTK) and Multiple EGF Like Domains 10 (MEGF10) and indirectly by inducing expression of complement cascade components in neurons (Schafer et al., 2013; Chung et al., 2013; Stevens et al., 2007). In the adult brain, astrocytes maintain neuronal and synaptic function, in part via many fine processes that ensheath neuronal synapses (Khakh and Sofroniew, 2015). Functions such as recycling neurotransmitters and buffering extracellular potassium to facilitate neuronal transmission (Allen, 2014) and taking up nutrients, including lipids and sugars from the blood, processing, and delivering them to neurons for energy (van Deijk et al., 2017). SAK3 administration to the TAF1 edited animals increased the number of GFAP-positive astrocytic cells relative to the TAF1 edited group and most likely restores these critical functions within the cerebral cortex.

In agreement with our previous studies, we found morphological abnormalities in cortical neurons in TAF1 edited animals (Janakiraman et al., 2019). In addition, we also observed a marked decrease in cell viability in cerebral cortex at layer 5. The plasticity of the pyramidal neuron morphology in motor cortex are influenced by the cognitive and executive functions (Jiang et al., 2020). To gain insight into the pathogenesis of these abnormalities, we investigated apoptosis. Apoptosis is the most common mode of neuronal death within the central nervous system (Kerr et al., 1972; Kerr et al., 2002). Indeed, we found that apoptosis was increased in TAF1 edited rats by TUNEL and cleaved caspase 3 analysis. Furthermore, we show increased levels of Bax in TAF1 edited animals. There is a plethora of evidence indicating a close relationship between Bax/Bcl-2 during apoptosis (Liu et al., 2011; Kerr et al., 2002; Green and Reed, 1998; Adams and Cory, 2007). Bcl-2 is the primary protein that inhibits cell apoptosis and is expressed in healthy cells, whereas Bax stimulates apoptosis by disrupting mitochondrial function (Adams and Cory, 2007). Hence, our observations suggest that TAF1 gene editing stimulates intrinsic apoptosis pathways in cortical neurons. Moreover, SAK3 treatment mitigates apoptosis caused by TAF1 gene editing.

To further gain insight into signaling pathways involved in the neuroprotective effects of SAK3 treatment against the adverse effects of TAF1 editing, we investigated the BDNF/AKT/GSK3β pathway. Brain-derived neurotrophic factor (BDNF) plays a key role in the pathogenesis of many neurodegenerative disorders (Giacobbo et al., 2019). BDNF binds to tyrosine kinase receptor B to activate Phosphatidylinositol-3-kinase (PI3K) and AKT, thus inhibiting GSK3β activity. We found that the neuroprotective effects of SAK3 treatment are associated with stimulation of in the BDNF/AKT/GSK3 pathway in TAF1 edited animals.

GSK3 has been reported to phosphorylate more than 40 proteins (Wada, 2009) and several studies highlight that GSK3 is an essential mediator of several signaling pathways important in cortical neuron homeostasis (Chen et al., 2008; Renaud et al., 2008; Nakamura et al., 2009). In the cell, two independently regulated GSK3 pools exist- the Wnt signaling pathway and the Wnt independent signaling pathway (Jaworski et al., 2019). Our results suggests that SAK3 treatment activates both pools of GSK3 since we observed an increased levels of CaV3.1 and FOXP2, which are downstream WNT signaling targets (Wisniewska et al., 2010; Bonkowsky et al., 2008). FOXP2 is the fork-head domain transcription factor, mutations of which have been associated with severe deficits in language (Lennon et al., 2007; Feuk et al., 2006; Zeesman et al., 2006). FOXP2 also regulates several downstream target genes that function in synaptic plasticity, neurotransmission, and neurite outgrowth (Vernes et al., 2007; Spiteri et al., 2007). In addition, we observed that SAK3 treatment restored the phosphorylation levels of CRMP2, at Thr509/514, a GSK3β substrate that is regulated independently of Wnt signaling and implicated in cortical neuron radial migration and dendritic orientation (Morgan-Smith et al., 2014). We conclude that regulation of GSK3β is critical for the disease modifying effects of SAK3 treatment.

In conclusion, our findings imply that the T-type calcium channels are novel molecular targets to develop therapeutics to treat TAF1 ID syndrome and that SAK3 is an attractive drug candidate to treat TAF1 associated neurological disorders.

## Supplementary Material

1

2

## Figures and Tables

**Table 1:** List of primary and secondary antibodies used in the study.

Abbreviation	Primary antibodies					
	Anti-	Host	Company	Catalog number	Application	Dilution

Bcl-2-associated X protein	Bax	Rabbit	Cell Signaling	14796S	WB	1:1000
B-cell lymphoma 2	Bcl2	Rabbit	Abcam	Ab196495	WB	1:1000
Brain-derived neurotrophic factor	BDNF	Mouse	Abcam	ab203573	IHC	1:500
					WB	1:750
Cleaved Caspase 3	C-Cas-3	Rabbit	Cell Signaling	9661S	WB	1:1000
Voltage-dependent T-type calcium channel subunit α1G	Cav3.1	Rabbit	Almone labs	ACC-021	IHC	1:500
Forkhead box protein P2	FOXP2	Rabbit	Abcam	Ab16046	WB	1:1000
Glial fibrillary acidic protein	GFAP	Rabbit	Dako	Z0334	IHC	1:500
phospho-Protein kinase B	p-AKT	Rabbit	Cell Signaling	9271S	IHC	1:500
Phospho-Collapsin response mediator protein-2	p-CRMP2	Sheep	Kina source	PB-043	WB	1:1000
Collapsin response mediator protein-2	CRMP2	Rabbit	Sigma	C2993	WB	1:1000
Phospho-Glycogen synthase kinase 3 beta	p-GSK3β	Rabbit	Cell Signaling	5558S	WB	1:1000
Glycogen synthase kinase 3 beta	GSK3β	Mouse	Cell Signaling	9832S	WB	1:1000
Class III β-Tubulin	βIII-Tubulin	Mouse	Promega	G7121	WB	1:1000

Anti-	Secondary antibodies					
	Label	Host	Company	Catalog number	Application	Dilution

Rabbit IgG	HRP-linked	Goat	Cell Signaling	7074S	WB	1:3000
Mouse IgG	HRP-linked	Horse	Cell Signaling	7076S	WB	1:3000
Sheep IgG	IgG (H + L)	Donkey	Jackson ImmunoResearch laboratories. Inc	AB_2340704	WB	1:20000
Rabbit IgG	Alexa 488	Goat	Life technologies	A11034	IHC	1:500
Mouse IgG	Alexa 488	Rabbit	Life technologies	A21204	IHC	1.500

WB = Western blot; IHC = Immunohistochemistry;

a Dilutions were from original stocks supplied by the vendors.
